# Spatial evolution and influencing factors of religious places from a socio-spatial perspective: An empirical analysis of Christianity in China

**DOI:** 10.1371/journal.pone.0265675

**Published:** 2022-03-21

**Authors:** Yujuan Chen, Ning Lin, Liang Ding, Jianping Qu, Qijun Zhou

**Affiliations:** School of Design and Architecture, Zhejiang University of Technology, HangZhou, China; Northeastern University (Shenyang China), CHINA

## Abstract

The mismatch between the supply and demand of religious places has resulted from a lack of management, population mobility, and urban socio-spatial differentiation. Furthermore, the identity of religious groups and the localized characteristics of their behavioral practices continue to dominate the evolution of religious places. Therefore, this study analyzed the spatio-temporal patterns of Christian activity places, and further studied the characteristics and mechanisms of various influencing factors on the spatial distribution of places using a geographically weighted regression model from a socio-spatial perspective. The following results were obtained: (1) Christian activity places form a distribution pattern of circling aggregation within the core area and polycentric dispersion outside the core area. There is a certain coupling relationship between spatio-temporal patterns of places and marketization in Yiwu City. (2) The spatial differentiation pattern of influencing factors has an obvious circle-layer characteristic. The regression coefficients of population mobility, age structure, public services, and jobs-housing space are larger, and social structure and spatial resource allocation are becoming the main driving forces of spatial differentiation. (3) Due to the differences in spatial resource allocation and believers’ behavioral choices, the above factors have distinct differences in urban core areas, urban-rural transition areas and rural areas.

## 1. Introduction

In the context of globalization, religious activities have become more closely linked to diverse social cultures, and the social relations and behavioral patterns of believers are becoming increasingly complex [1]. Furthermore, the creation of religious spaces is constantly testing governments’ responses to urban cultural and religious governance and related spatial development [2, 3]. Therefore, the coordination between the spatial distribution of religious places and urban development has become an important issue.

Currently, research on urban public service facilities tends to focus on location choice, allocation standards and evaluation systems, as well as spatial differentiation patterns and equity [4–6]. However, it is worth noting that as religious places are special kinds of public service facilities, they do not fall directly under governments’ macro-control; instead, their establishment follows a bottom-up approach that stems from the believers themselves [7–10]. Therefore, it is necessary to consider the spatial effect of religious groups emphatically, and to try and understand the mechanism behind the spatial patterns of religious places from the perspective of social and space interaction.

To this end, the current research is mainly divided into two aspects: material characterization and social interaction. In terms of material characterization, scholars have primarily studied the spatial evolution characteristics of religious places through a spatial analysis method, or they have explored microscopic changes in religious architecture through field investigation. For example, in his research on the evolution of Mormon spaces, Meinig found that they have a “core-territory-periphery” structure [11], whereas Chiodelli et al. found that religious places took different forms across time depending on the specific religious architectural space [12]. Secondly, some studies have made preliminary discussions on the factors that influence the spatial characteristics of religious places from different perspectives, but due to the complexity of religion, the research results are often fragmented. For example, Zelinsky found that there were different spatial differentiation patterns in various religious places, and that the region and church were the main reasons for this phenomenon [13]. Xue et al. pointed out that the spatial distribution of Christian churches is mainly affected by traffic location and population factors. In addition, some scholars proposed that the process of secularization and the diversity of religions determine construction of religious places to some extent [14–16]. In terms of social interaction, based on the background of regional mobility, most studies have focused on the influence of believers’ diverse social interactions of religious groups on the shaping of urban religious places. For example, Hatziprokopiou et al. pointed out that in order to change their marginalized social and economic status, Muslim transnational immigrants in Greece committed themselves to establishing of a central mosque in Athens, the capital, in order to gain respect and recognition in Greek society [17]. Rejecting and ignoring the local society and seeking to establish new social relations are the main demand of believers [18, 19]; at the same time, these studies have also stressed that the construction, renovation, and relocation of religious places and varied spatial evolution occurred as a result of the economic improvement of religious groups and changes in their residential areas [20–22].

From this context, it is clear that religious groups play a role in the production of religious spaces through complex and interactive behavioral practices and interactive that occur as a result of different religious and cultural identities, as well as local attachments [23–26]. Furthermore, religious places have become important symbols of spiritual expression and areas for believers to socially participate [27]. From the perspective of socio-spatial theory, this socialization process is continuous. This theory holds that society and space are dynamic and influence each other. Social space, as the materialization of social existence, is a projection of social relations formed in space through actual activities [28]. Social relations determine the characteristics of space, and space itself shapes and reproduces social relations [29].Furthermore, as religion is itself social and its function is often realized through belief ceremony, the material place plays an important role. Therefore, the production of religious places can also be regarded as a continuous socialization process.

In summary, there are some gaps in the traditional research on spatial characteristics and the influencing factors of religious places. On the one hand, many studies have separated the material characterization of religious places from social interaction, and take their respective aspects as the starting point of research; this makes it difficult to comprehensively explore the establishment of religious places. On the other hand, locality plays an extraordinary role in the formation of identity and place attachment [30], which leads lead to great differences in religious places across regions. Such studies have lacked a quantitative method that can adequately interpret this spatial heterogeneity, thus affecting their judgment of influencing factors. Therefore, this study employed quantitative research methods such as kernel density estimation and Geographically Weighted Regression models to explore the spatio-temporal distribution patterns of religious places, and identified the spatial heterogeneity of social and material factors. From a socio-spatial perspective, it revealed the internal mechanism of the spatial evolution of religious places., with a view to providing theoretical guidance for the development of religious work and related policy formulation.

## 2. Materials and methods

### 2.1. Overview of this study area

The study area is in Yiwu City, Zhejiang Province, China, which has an area of about 1,105 km^2^ and 14 subdistricts (towns) under its jurisdiction. As an important node city in the construction of “The Belt and Road Initiative,” and the world’s largest distribution center for small commodities, Yiwu City had a local population of 818,000 and an immigrant population of 1,429,000 in 2018. The registered foreign population originated from 195 countries and regions worldwide, amounting to 551,000 people, whereas the resident foreign population totals 13,000 people. As the population structure is complex and highly mobile, it contains multiple religious clusters.

**Fig 1:**
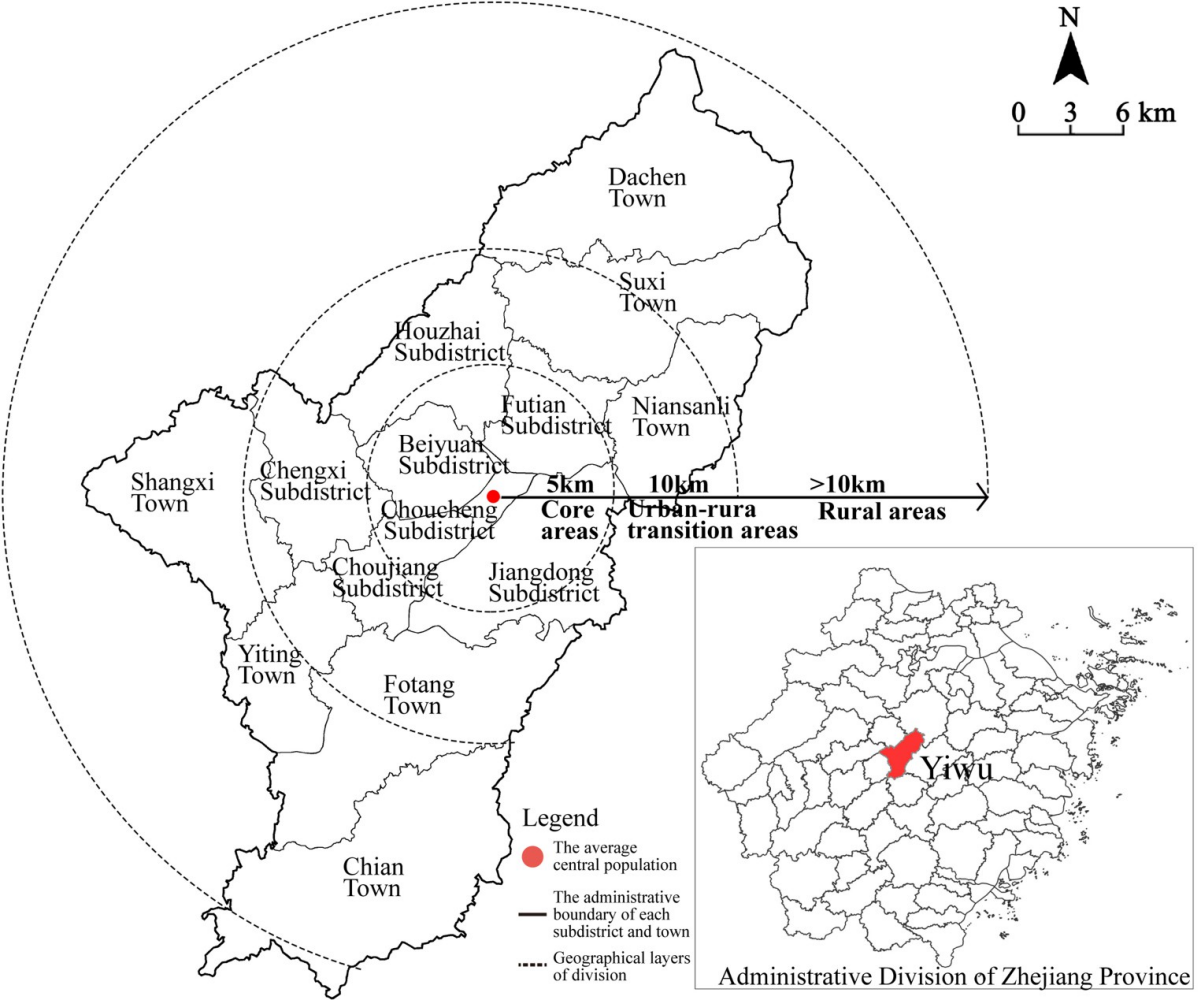
Research scope and layers of division. With the average central population of Yiwu city as the center, the study area was divided into three circles: core area, urban-rural integration area and rural area. Source: created by the author based on the base map of Yiwu which comes from the Open Street Map (OSM) geographic data platform (https://www.openstreetmap.org/).

This study focused on Christian activity places including churches and other fixed places of religious activities. Christianity is characterized as having a “low, small, scattered, high frequency, and rapid spread” in Yiwu city, and many private congregations still exist in areas with frequent population movements. Church members generally come from the surrounding communities, with significant community based characteristics in spatial distribution [31]. As Yiwu reflects the diversity of religions present in China, it was chosen as the place to conduct such an empirical study. In order to facilitate a comparative analysis based on concentric zone theory and combined with the situation of local land use [32, 33], this study used the average central population in Yiwu as the center of the circle. The area within the 5 km radius is the urban core area, the area within 5–10 km is the urban-rural integration area, and the area beyond 10 km is the rural area (Fig 1).

### 2.2. Selection of the index system

The formation of the spatial pattern of Christian places is the result of multiple interactions and intersections between various factors. Based on the theoretical analysis framework, considering the accessibility and quantifiability of indicators and eliminating the relevant variables with multiple covariates, the following influencing factors were selected as independent variables (as shown in Table 1): (1) Socio-economic attributes: on the one hand, this study considered three aspects—population density, age structure, and population mobility. Population density reflects the distribution of believers and affects missionaries preaching tendencies [34]; People of different ages have different views on religion due to different attitudes and life pressures [35]; In the context of regional integration, migrant populations long for a sense of belonging, and Christianity provides them with a place for this [27]. On the other hand, the level of overall development was incorporated into the index system. Areas with higher levels mean that there is more overlap amongst most diverse populations, thus providing a large number of potential believers for churches [20]. (2) Physical spatial environment: the degree of transportation convenience is the most important consideration in the selection of missionary places [36]. This study used road network density within the grid to indicate this characteristic variable. The spatial distribution of the mobile population is closely related to the support of basic service facilities, employment space, and inhabitation space; as the basic spatial carrier for urban residents to conduct social activities, it influences the spatial distribution of the believing masses in spatial production [37].

**Table 1:** The characteristic variables that influence spatial differentiation of Christian places.

Variable classification	Explanatory variable	Variable description
Socio-economic factors (S)	Population density (S1)	Population size per unit area of land
	Population ageing (S2)	Proportion of population aged 65 and above in total population
	Population mobility (S3)	Proportion of migrant population to urban population
	Level of overall development (S4)	Per capita GDP
Physical environment factors (P)	Degree of transportation convenience (P1)	Total length of road network/sample unit area
	Basic service facilities(P2)	The average density of POI for medical, educational and commercial facilities in the sample unit
	Employment space (P3)	Average density of POI of the company in the sample unit
	Inhabitation space (P4)	Average kernel density of resident POI in the sample unit

### 2.3. Data sources

This study used the following two types of data (as shown in Table 2). The socio-economic data were obtained from the statistical yearbook of Yiwu City in 1990, 2000, 2010 and 2018 as well as the annual statement of population statistics. The geospatial data included (1) data on Christian activity places, which were provided by Yiwu Citizen’s Religious Bureau and supplemented by actual research; (2) influencing factor data, which were based on the data of Landsat 5, Landsat 7 and Landsat 8. Remote sensing images were preprocessed with radiometric correction, geometric correction, and image registration, and the land use data in 1990, 2000, 2010 and 2018 were obtained by combining supervised classification with visual interpretation, and the rural settlement data were extracted. The POI (Point of Interest) of communities and apartments in the central city were integrated to comprehensively represent the inhabitation space of Yiwu City. Remote sensing images were taken from Landsat Science (http://landsat.visibleearth.nasa.gov/); the POI of companies was characterized the employment space; the POI of hospitals, schools, commercial facilities, and other basic services were characterized as basic service facilities. The POI and road network data were crawled from the Gaode Map geographic service platform (https://lbs.amap.com/). (3) In this study, the map of Yiwu city was taken from the Open Street Map (OSM) geographic data platform (https://www.openstreetmap.org/). OSM aims to provide users with free and easy-to-access digital map resources, which is currently the most popular spontaneous geographic information platform.

**Table 2:** Data sources and description.

Data source	Data description
Yiwu Citizen’s Religious Bureau	Data on Christian activity places(51 in total)
The statistical yearbook of Yiwu City in 1990, 2000, 2010 and 2018	Population density/ Proportion of population aged 65 and above in total population / Per capita GDP
The statement of population statistics of Yiwu City	Proportion of migrant population to urban population
the Gaode Map geographic service platform	Road transport network/ POI of residence, companies, hospitals, schools, commercial facilities, recreation, sports and green park(8925 in total) https://lbs.amap.com/
the Open Street Map (OSM) geographic data platform	The map of Yiwu city https://www.openstreetmap.org/
Landsat Science	The land patches of rural settlements http://landsat.visibleearth.nasa.gov/

### 2.4. Research method

#### 2.4.1. Kernel density estimation

The kernel density estimation method can accurately demonstrate the spatial distribution and agglomeration of point data [38], so it was used to analyze the spatio-temporal distribution pattern of religious places. The kernel density estimation *g*(*x*) is used to estimate the overall point and line densities by using moving cells through the following equation:

$$g(x)=\frac{1}{sh}\sum_{i=1}^{s}k(\frac{x-x_{i}}{h})$$ (1)

Where *x_i_* represents the coordinate position of point *i, i* = 1, 2, 3…, *s*; *s* is the number of coordinate points; *h* represents bandwidth; and *k* is a weight function that is used to estimate the number and utilization of data points. Based on field questionnaire interviews and the service radius, the bandwidth was set to 3km in this research.

#### 2.4.2. Geographically weighted regression

The socio-spatial transformation process of religion has an obvious localization phenomenon and shows spatial heterogeneity. To overcome the limitations of traditional linear regression models in the estimation of spatial characteristics, the Geographically Weighted Regression (GWR) model was used to measure the influence of various factors on the spatial distribution of Christian places across different regions, thereby revealing the complex relationship between the study objects and spatial influence factors. The relationship varies depending on the spatial location, and the results are more aligned with objective reality [39]. The model is as follows:

$$y_{i}=\beta_{0}(m_{i},n_{i})+\sum_{j=1}^{n}\beta_{j}(m_{i},n_{i})x_{ij}+\varepsilon_{i}$$ (2)

Where (*m_i_, n_i_*) represent the central geographic coordinates of the *i*-th study unit; *β_i_*(*m_i_, n_i_*) is the value of the variable continuous function *x_ij_*(*m, n*) in the *i*-th county unit; and *ε_i_* is a random term. In this study, the dependent variable was the average kernel density value of Christian places, and the independent variables were divided into two categories: socio-economic and material space. (1) The data of socio-economic variables were restricted by statistical cells, and subdistricts (towns) were used as the smallest spatial cell; (2) The material space variables were divided into grids (500 m×500 m), using the fishing net tool to make the analysis results more accurate, and each grid was given a variable value. The grids with sparse sample points were removed, and 3,459 study cells were obtained in total. The two types of independent variables and the corresponding dependent variables were put into the GWR model for estimation; the Gaussian function was chosen as the weight function and the cumulative Akaike Information Criterion (AICc) was used as the bandwidth determination criterion.

To avoid having results that are biased by the interaction between indicators, the Variance Inflation Factor (VIF) was first applied to the eight indicator variables (listed in Table 1) using SPSS software that tests for multicollinearity. As the VIF of each variable was less than 5, there was no multicollinearity between variables. According to the ordinary least squares (OLS) model fitting results, the two types of variables reached a highly significant level (p < 0.01) and the model fit was good at 90.8% and 78.1%, respectively. This can better explain the distribution pattern of Christian places. Before using model estimation, this study calculated global autocorrelation indices using GeoDa software, and measured Moran’s I index values of 0.509 and 0.639, respectively. Both passed the significance test at the 0.01 level, indicating that the dependent variable itself possessed significant positive spatial correlation. The GWR model bandwidth was calculated using MGWR software, and the model fit was 92.2% and 99.6%, respectively (Table 3), both of which are better than the OLS model results. The decreasing values of AICc all exceeded 3, which proved that the GWR local model fit was better and could more effectively explain the influence situation of the independent variable.

**Table 3:** Test result of the GWR model.

Model parameter	Socioeconomic factors(S)	physical environment factors (M)
Bandwidth/Neighbors	14	1462.570
ResidualSquares	4.381	1.230
EffectiveNumber	7.849	1430.372
Sigma	0.844	0.023
AICc	65.971	-15384.357
R^2^	0.962	0.998
Adjustment R^2^	0.922	0.996

## 3. Result

### 3.1. Spatio-temporal characteristics of the evolution of religious places

**Fig 2:**
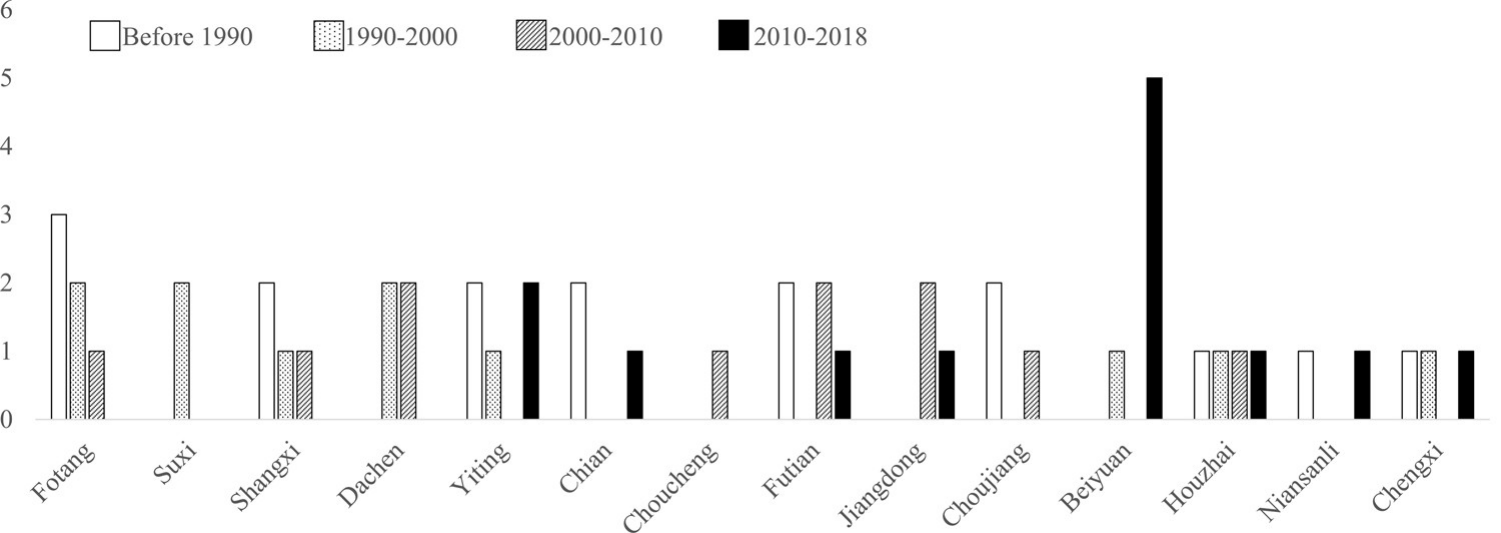
The increased number of Christian places in each subdistrict (town) of Yiwu over time. Here, we show the growth of Christian places in Yiwu city in four time periods from 1990 to 2018.

**Fig 3:**
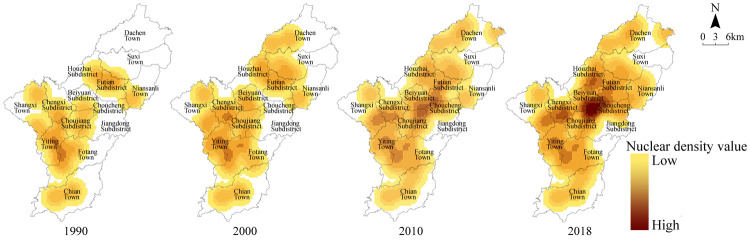
Kernel density evolution of Christian places in 1990, 2000, 2010 and 2018. The kernel density estimation method was used to analyze the spatio-temporal distribution pattern of Christian places. Areas with high kernel density indicate that there are more Christian places distributed. Source: created by the author based on the base map of Yiwu which comes from the Open Street Map (OSM) geographic data platform (https://www.openstreetmap.org/).

Yiwu City now has 51 Christian places. In terms of numbers, Futian, Beiyuan, and Fotang currently have a higher distributions. The rising trend of the number of places within the subdistricts is more obvious than that of towns (Fig 2). In order to visualize the spatial evolution of places, the kernel density estimation of the spatial distribution of Christian places in 1990, 2000, 2010, and 2018 was conducted. This shows that the density center of spatial distribution gradually shifteds from the south to the center, and the spatial polarization characteristics increased significantly, forming a distribution pattern of “block-like circles gathering within the core area and polycentric dispersion outside the core area” (Fig 3).

In 1990, the high value kernel density area was concentrated in Fotang Town and Yiting Town, while the northern area was scarce, showing an obvious imbalance in the north-south distribution. In 2000, the kernel density value increased in the central and northern regions, and new gathering points were formed in the junction area of Chengxi Subdistrict and Choujiang Subdistrict. In 2010, the kernel density value of the central area was at the highest level. Fotang -Yiting area and the Chengxi-Shangxi area also emerged as gathering points. In 2018, the central urban area became the area with the highest degree of agglomeration. The internal circle was formed in the Futian, Choucheng, and Beiyuan subdistricts that constituted the center and spread to the periphery in a stepwise manner. At the periphery of the core area, Fotang-Yiting, Chengxi-Shangxi, and the Houzhai subdistrict formed three nodes with the next highest value, which were scattered in the shape of an “extreme core”. From 1990–2000, the degree of spatial agglomeration of places in Fotang-Yiting area increased, while the northern area began to show a slow upward trend. From 2000–2010, the central area gradually became the new core agglomeration area, showing a “core-edge” distribution characteristic. From 2010–2018, Christian activity places continued to gather at the center, while the degree of agglomeration at each gathering point rose. Comparing the four time points revealed that the spatial distribution of Christian places had been spreading from the south to the central and northern areas, gathering in the central city and some townships, and decaying diffusely from the center to the periphery.

The spatial evolution of Yiwu’s Christian activities is inextricably linked to the development of the city’s spatial structure, and as a market-driven city, the relationship between the trade town and the city is the most important factor in its spatial structural change. Since 1982, Yiwu has experienced five generations of market development and is currently moving towards the sixth. The interaction between the trade town and the city has gone through four stages of “integration, divestment, separation, and mixing.” In the context of industrialization and urbanization, which is driven by marketization, the multiplier effect of market agglomeration in Yiwu has led to the spatial agglomeration of the population. As the spread of Christianity depends on the influence of demographic factors, several new places have been created based on its path dependence and population mobility. Therefore, the population of believers under marketization reflects the characteristics of semi-urbanization and mobility, and there is a complex relationship between believers and urban space. It is necessary to further analyze the main factors affecting the spatial distribution of Christian places from the perspectives of population, development level, and physical space, and to explore whether there are spatially divergent characteristics within each factor.

### 3.2. Analysis of the factors influencing the spatial pattern of religious places

#### 3.2.1. Characteristics of the role of factors influencing socio-economic attributes

**Fig 4:**
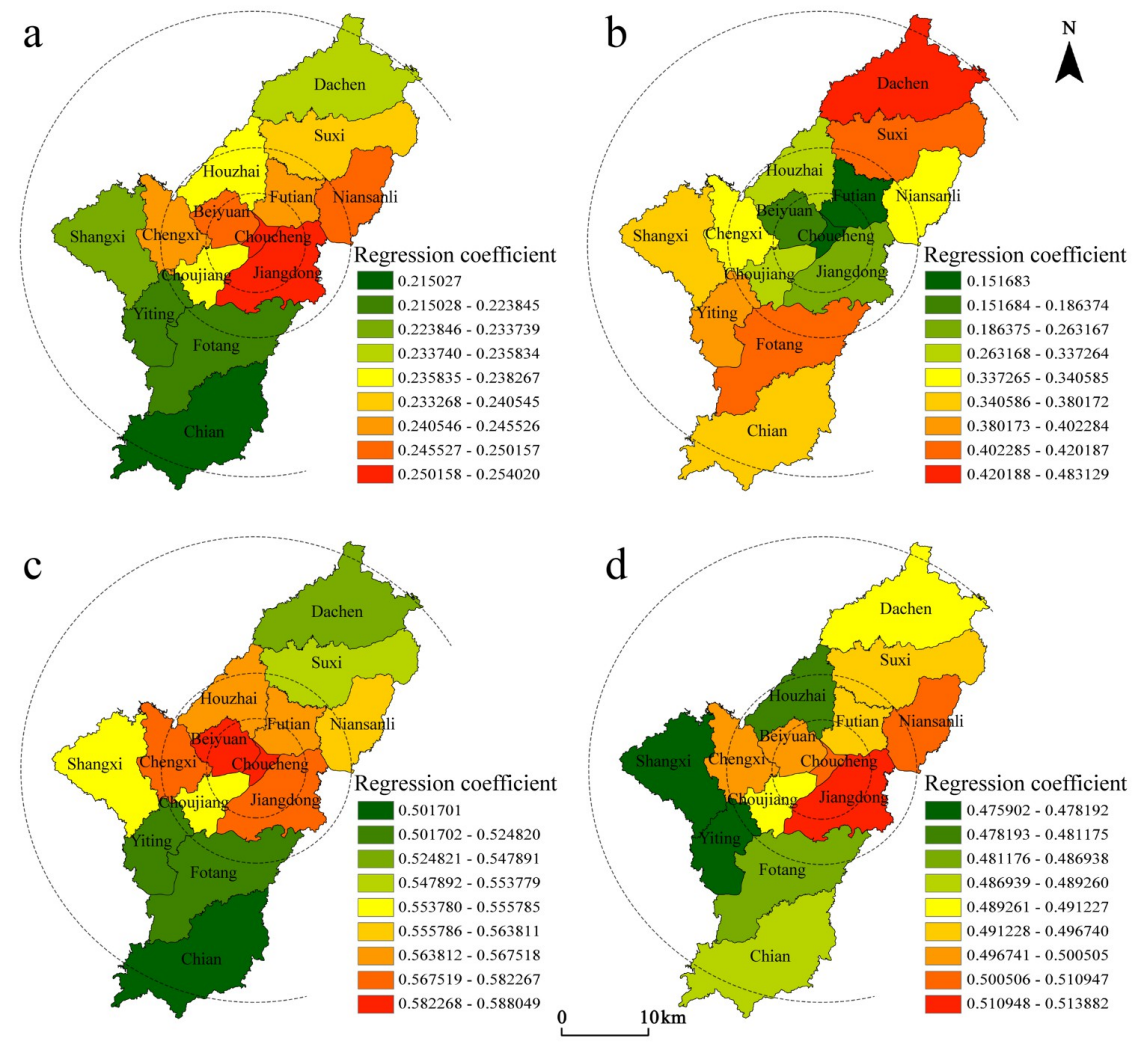
Spatial differentiation of factor for socioeconomic attributes. (a) the spatial distribution of regression coefficients of population density; (b) the spatial distribution of regression coefficients of population ageing; (c) the spatial distribution of regression coefficients of population mobility; (d) the spatial distribution of regression coefficients of overall development level. Source: created by the author based on the base map of Yiwu which comes from the Open Street Map (OSM) geographic data platform (https://www.openstreetmap.org/).

As shown in Table 4, the ranking of the absolute values of the regression coefficients among the socio-economic factors, which was based on the mean, was population mobility (S3) > overall development level (S4) > population aging (S2) > population density (S1); as the regression coefficients were all positive, this indicates that each factor had a posi-tive driving effect on the distribution of Christian places, and that there is spatial var-iability (Fig 4).

**Table 4:** Calculation result statistics of the GWR model for socioeconomic attributes.

Variable	Minimum	Lower quartile	Median	Upper quartile	Max	Mean	Significance
Population density (S1)	0.215	0.231	0.239	0.249	0.254	0.239	***
Population ageing (S2)	0.152	0.236	0.359	0.404	0.483	0.327	***
Population mobility (S3)	0.502	0.542	0.560	0.579	0.588	0.556	***
Level of overall development (S4)	0.476	0.485	0.494	0.503	0.514	0.494	***

Note

*** is 0.1% significance level.

1. Influence of population density: The regression coefficients were all positive, indicating that the higher the density of population distribution, the denser the distribution of Christian places; this is in line with previously established research findings. The influence of population density on the spatial distribution of Christian places showed an overall trend of decreasing from the core area to the outer circle, indicating that the more inward the circle, the stronger the role of population density. The high value areas are mainly concentrated in Jiangdong, Choucheng, Niansanli and Beiyuan subdistricts, which rely on industrial clusters to attract many foreigners for employment, including foreign believers, that indirectly affect the distribution of Christian places. The influence coefficient of rural areas was low, indicating that the concentration of the population in rural areas has a small impact on the distribution of Christian places.
2. Influence of population aging: The regression coefficient formed a zone of low values in the central city, and the coefficient in the northern and southern zones was significantly higher than that of the central zone. This indicates an obvious urban-rural difference in the force of this factor on the distribution of Christian places, which is more significant than other factors (Fig 4B). The high proportion of foreign workers in the central city leads to a clear trend of younger believers, while rural believers are mostly elderly people that are more affected by aging, indicating that there are large urban-rural differences in the degree of influence of the aging factor on the spatial distribution of Christian places based on generational differences.
3. Influence of population mobility: The high value of the regression coefficient was concentrated in the Choucheng and Beiyuan subdistricts, followed by Jiangdong, Chengxi, Futian, and Houzhai subdistricts in the periphery, indicating that Christian believers account for a relatively high proportion of the mobile population in the above areas. With regional integration and market internationalization, interregional population movement will be further strengthened, and the central city, as the socio-economic and cultural center of the city, will become the main destination for believers. Meanwhile, the regression coefficient of Choujiang subdistrict was significantly smaller than other subdistricts, indicating that there is also spatial heterogeneity in the effect of mobility factors within the central city. This is closely related to the cost of moving in (e.g., schooling, housing prices, transportation, labor market segmentation, and policy preferences), thus affecting believers’ willingness to do so. The coefficient of regression was lower in the northern and southern regions, which are subject to a relatively low net inflow of people under urban-rural mobility for rural areas. Therefore, the influence of population mobility has a weaker impact on the distribution of Christian places in northern and southern regions, such as Dachen, Yiting, Fotang and Chian Town.
4. Influence of overall development level: The regression coefficients were all positive, indicating that the level of economic development has a positive driving effect on space in the whole region. The economic development will make different cultures more inclusive in a local area, and provide the material basis and cultural atmosphere for the spread of Christianity. The high-value areas within the core region were concentrated in Jiangdong and Niansanli subdistricts, which are linked with development in Dongyang City, as well as labor, capital and other factors that influence the inflow of believers. The Choujiang subdistrict, which is also located in the core area, had a relatively low regression coefficient compared to the old city center. The reason for this is that economic development depends on the input of urban land resources to some extent. However, when land urbanization stabilizes, the marginal benefit of the level of economic development on the distribution of places will diminish. Although the old city has a strong economic base, the tendency of believers to choose the location of religious activities does not depend largely on its economic level.

In general, socio-economic factors have a positive driving effect on the spatial distribution of Christianity within the core area of Yiwu City, and the forces of each factor showed obvious circling characteristics, with population density, population mobility, and overall development decreasing from the inside to the outside, whereas the level of aging had the opposite trend. At the same time, the forces of each factor showed different spatial patterns in different circles, which are related to the differences in urban socio-economic development.

#### 3.2.2. Characterization of the role of factors influencing the physical space environment

**Fig 5:**
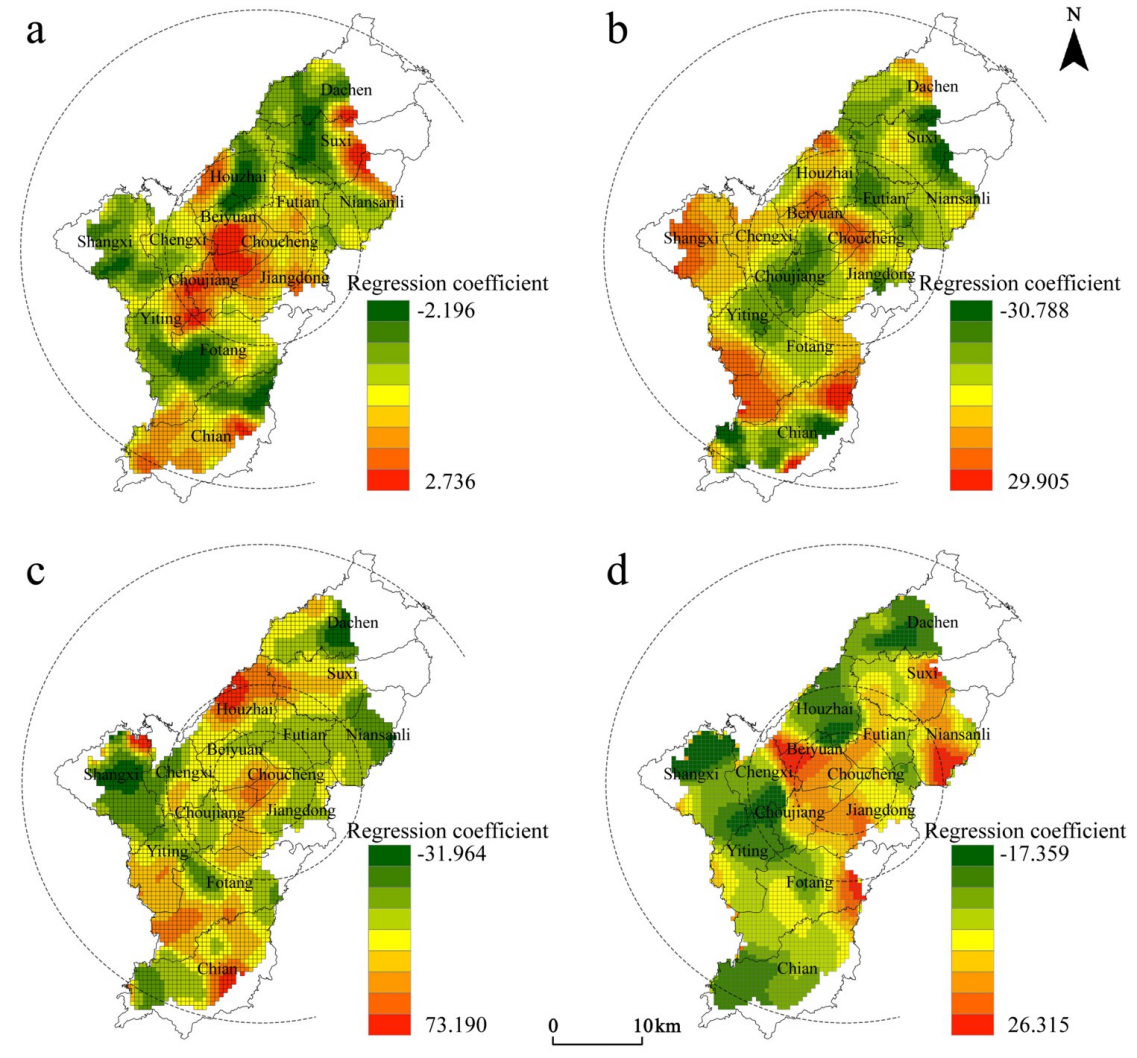
Spatial differentiation of factor for physical space environment. (a) the spatial distribution of regression coefficients of transportation convenience; (b) the spatial distribution of regression coefficients of basic service facilities; (c) the spatial distribution of regression coefficients of employment space; (d) the spatial distribution of regression coefficients of inhabitation space. Source: created by the author based on the base map of Yiwu which comes from the Open Street Map (OSM) geographic data platform (https://www.openstreetmap.org/).

As shown in Table 5, the average magnitude of global influences on the physical spatial environment was ranked as follows: basic service facilities (P2) > employment space (P3) > inhabitation space (P4) > degree of transportation convenience (P1), with the respective variables showing a spatially non-stationary relationship with the dependent variable (Fig 5).

**Table 5:** Calculation result statistics of the GWR model for physical space environment.

Variable	Minimum	Lower quartile	Median	Upper quartile	Max	Mean	Significance
Degree of transportation convenience(P1)	-2.196	-0.137	0.031	0.268	2.736	0.077	***
Basic service facilities(P2)	-30.788	-0.439	0.644	2.317	29.905	1.312	***
Employment space (P3)	-31.964	-1.083	0.109	1.223	73.190	1.077	***
Inhabitation space (P4)	-17.359	-0.355	0.553	1.697	26.315	0.755	***

Note

*** is 0.1% significance level.

1. Influence of traffic convenience: As depicted in Fig 5A, the high value of the regression coefficient of traffic convenience in the core area was concentrated in the Jiangdong, Chucheng, and Beiyuan subdistricts, most of which are old urban areas with perfect supporting facilities that are more influenced by traffic. The influence of traffic in the urban-rural transition area showed polarized characteristics, and the high value areas are distributed across Futian subdistrict and Fotang-Yiting area. This indicated that under the “point-axis” spatial development model, Yiwu’s transportation coincides with the development of industries or supporting facilities, and believers participate in the socio-economic activities of the region as a migrating population. The role of transportation convenience in the township areas on the location choice of Christian places was smaller than that of central urban areas, and the regression coefficient was negative in some township areas. Some studies show that convenient transportation conditions may also make it less necessary to build new churches [40], and this interconnection of infrastructure may further contribute to population mobility between rural and urban areas, leading to a decrease in believers in rural areas.
2. Influence of basic service facilities: As illustrated in Fig 5B, the areas with high values of regression coefficients in the core area were mainly distributed in Choucheng, and a negative area appeared in the south, thereby showing significant spatial differentiation characteristics. The public service supply capacity of the core area has been enhanced as the city’s capacity has been continuously upgraded. However, the access threshold has also been increasing, resulting in a spatial mismatch between supply and demand for the citizenship of the floating population in the core area as a gathering place, causing some believers to leave the area due to high living costs. The regression coefficients within the urban-rural transition area also had negative values except for Houzhai subdistrict. In other words, the basic service facilities in this area effectively dampen the distribution of Christian places.
3. Influence of employment space: As shown in Fig 5C, the regression coefficient within the core area decreased from the center to the outer circle; the high value areas of regression coefficient within the urban-rural transition area were scattered within the circle in the form of polar nuclei. These areas are key to Yiwu’s industrial layout, relying on manufacturing employment opportunities that attract mobile believers to move in. The high-value rural areas are distributed across the more industrially developed Suxi town and Fotang-Yiting area.
4. Influence of inhabitation space: As presented in Fig 5D, the regression coefficients within the core area were all positive. The areas with the highest value were Beiyuan, Futian, Niansanli, and Jiangdong subdistricts, with an overall decrease from the core area to the periphery. A comprehensive comparison of Figs 4C, 5C and 5D showed that this factor is more consistent with population mobility in terms of spatial characteristics, but there is a misalignment between the above two and the employment space factor within the core area. The phenomenon can be explained by the fact that the high-value area of the residential and mobile population factors is the living support area of the professional market in Yiwu City, where the land cost is low due to relatively high land development intensity. As the corresponding residential cost is not high, which attracts more believers to move in, a misalignment is created between believers’ employment and residence. The significant negative value appeared at the edge of the rural area, which is, to some extent, the result of evacuating villages in Yiwu as rural land use intensified.

### 3.3. Mechanisms of the spatial formation in the evolution of religious places

Based on socio-spatial theory, the behavioral activities and social relations between believers play a direct role in the construction of urban elements and places of Christian activity. In other words, locality is key to the group’s identity and behavioral activities. The mechanisms of spatial formation act very differently between urban and rural or core and suburban areas due to different geographical characteristics. Therefore, because of the pattern of spatial differentiation in the circling of Christian activity places, it was necessary to explore the influencing factors in different circles.

#### 3.3.1. Core areas: Identity and class divisions

Spatial differentiation within the core area, which is based more on housing and community, exacerbates the fragmentation and stratification of urban social space [41], and the impact of corresponding policy factors (i.e., school district system, subsidized housing) further exacerbates population differentiation [42]. First, public resources begin to cluster in high-end neighborhoods, which ultimately means that some communities are deprived of communal spaces and social opportunities [43]. Second, the secularization of local religions, ethnic cultures and identities are continuously reproduced in religious spaces, and the growing middle or emerging social classes in Yiwu make up a certain proportion of believers. The attraction of the church for upwardly mobile people from working-class backgrounds makes them an organizational group with a strong sense of cohesion [44]. The core area of Yiwu has a cluster of Wen merchants, who have formed “karmic” settlements based on hometown business ties and have reproduced a number of Christian activity places that specifically serve Wen merchants. These mobile faith groups in the urban core tend to homogenize in terms of residence, production, and consumption patterns that are based on local socio-spatial differentiation and ethnic identities. As believers build internal interaction spaces with each other, this embeds Christian places into the urban space in the form of spatial differentiation.

#### 3.3.2. Urban-rural transition areas: Subject behavior and value dispositions

This area is the main position of urban spatial expansion. With early spatial disorderly growth that is characterized by single land function and morphological fragmentation, the phenomenon of co-existing industrial areas, new towns, and urban villages will appear. The diversity of the residential population leads to complex changes in religious beliefs. In the context of a polycentric spatial structure that gradually replaces the monocentric development model [45], many new areas have been planned around the central city of Yiwu. Thus, the original accumulation of rural believers in the suburbs and the mobile believers under urban-rural integration are intertwined; additionally, the gap between believers caused by having different priests complicate overall Christian faith pattern. This background has led to a pluralistic reproduction of the social space of faith communities, with well-educated and socially resourced believers who have higher expectations of doctrinal parsing and faith practice, while most of the groups who have settled through work are faced with livelihood problems and follow a simpler form of worship. Consequently, believers that have different behavioral values are distributed within the urban-rural transition area. Furthermore, the spatial reproduction of places for Christian activities is realized in accordance with certain laws in response to multicultural job-housing spaces.

#### 3.3.3. Rural areas: Older age groups and native organizations

The development of Christianity is rooted in rural communities, and the development of rural Christian churches began because of the psychological comfort sought by middle-aged and elderly people with a low level of education and an absence of spiritual life. In addition, the fear of illness and death perpetuated this need, as the existing security system was inadequate. Subsequently, Christianity interacted with the social process of self-organization in the countryside, which is a typical social network of acquaintances, that have poor social mobility and strong homogeneity among them. There exists a native type of vernacular community faith organization [46] that is characterized by the spread of faith through kin, friends, and neighbors. However, as rural areas began the pattern of shrewd contraction [47], the original kinship network in the countryside diverged into individual small families. The vernacular cultural elements were no longer the force that coordinated the community order, and the administrative system and modernization mechanisms constructed a rigid new community order [48]. In this new context, Christian places in rural areas become more influenced by policy factors and are more prone to becoming marginalized or even disappearing in the new community development plan; this grows as the influence of socio-economic, as well as physical and spatial factors, intensifies.

**Fig 6:**
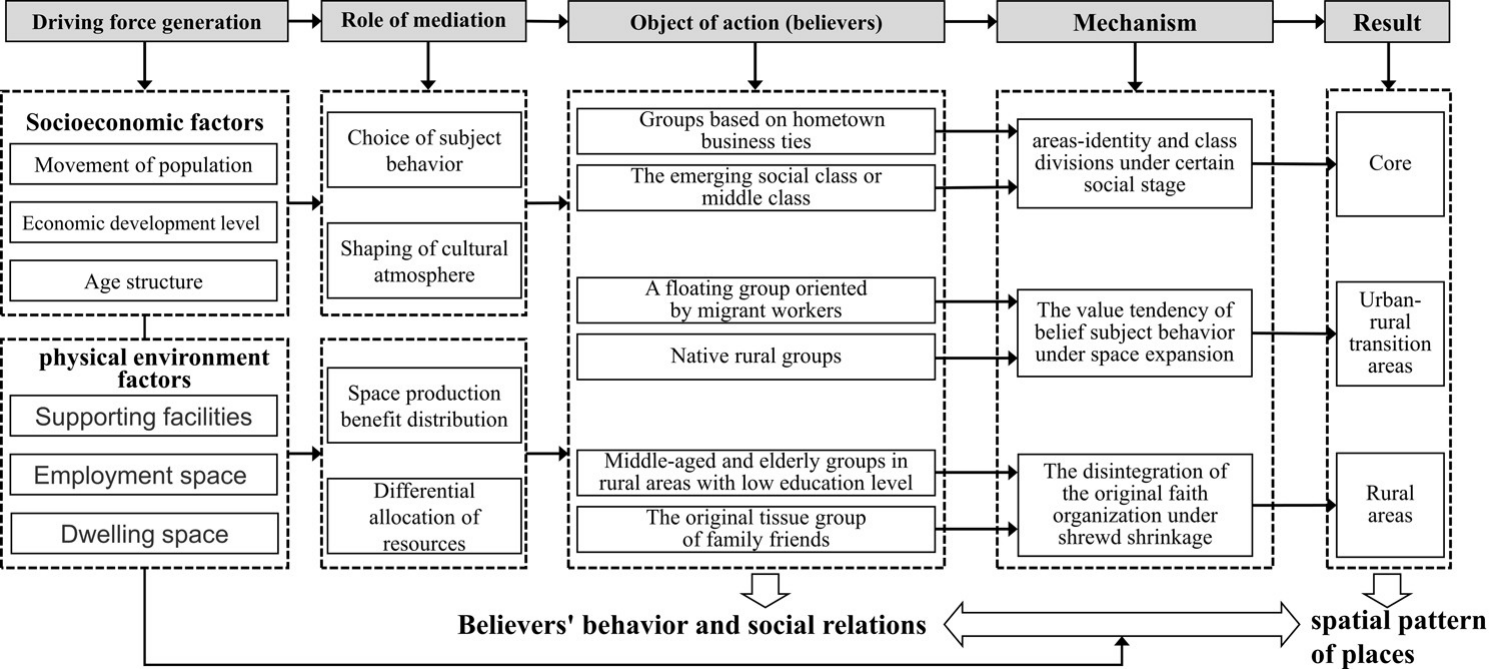
The process of influencing factors in the spatial differentiation pattern. The whole process includes five parts: first, the driving force includes socioeconomic factors and physical environment factors. Secondly, under the action of the above two kinds of factors, two mediations are created respectively: believers’ behavioral choices and Spatial resource allocation. Thirdly, three types of typical object are formed. Fourth, different categories of object follow different mechanisms. Finally, the characteristics of circle layer differentiation are formed in space.

Under the combined response of different action mediators, the two types of influencing factors will act on different subjects of the religious masses, which cause different action processes in different areas, and finally realize the socio-spatial interconversion, leading to the formation of a circle-like spatial differentiation pattern (Fig 6).

## 4. Conclusion

Taking Christian activity places in Yiwu as an example, this study used a variety of spatial analysis methods and spatial econometric models to explore the spatio-temporal patterns, the role of influencing factors, and the mechanism of action. The main conclusions are as follows.

1. Evolution of spatial pattern: From 1990–2018, the distribution of Christian activity places in Yiwu City continuously spread from the southern to the central and northern parts of the city, eventually forming a spatial pattern of circling aggregation within the core area, polycentric dispersion outside the core area, and diffused decays in the periphery. The formation of this pattern is closely related to the development of industrialization and urbanization, which was driven by the marketization of Yiwu City.
2. The role of influencing factors: In the distribution of religious places, all the influencing factors had a circular pattern of spatial differentiation. Among them, the regression coefficients of the factors of population mobility, age structure, public services, and job-housing spaces are larger, indicating that the social factors of population and spatial resource allocation have become the intrinsic driving force in the formation of the spatial pattern of Christian places.
3. The mechanism of action of the influencing factors: In the context of material space reconstruction and social and economic transformation of the times, the above influencing factors will lead to the differential allocation of resources and the diversification of the believers’ behavioral choices, which have different effects on religious people in the core areas, urban-rural transition areas and rural areas of Yiwu City; this further drives the spatial reproduction of Christian activity places and eventually results in a circular differentiation pattern.

## 5. Discussion

### 5.1. Study innovations and significance

To supplement the existing findings in the contexts of China and other countries, this study offers the following findings:

1. Innovation of method and perspective——From the perspective of urban planning, previous studies were limited by their own discipline and tended to study space from a single perspective. In addition, the research method had limited functionality and could not effectively measure the relationship between variables. Based on these limitations, this study contributed two innovations to the field. Firstly, as a method to identify spatial heterogeneity, the GWR model was used as it can incorporate spatial factors into the measurement, discover the causal relationship and the closeness of the relationship between two variables, and intuitively present the research results quantitively and visually. For religious places with local characteristics, this method is highly applicable. Secondly, the layout of religious places largely depends on the migration of believers, and the identity and behavioral characteristics of the crowd are key to the layout of religious places. Social-spatial theory provides an innovative and appropriate research perspective from which this can be investigated. Focusing on the interactive relationship between people and space, it can help this study explain the formation mechanism of the spatial pattern of religious places, so as to explore the rules of the layout in more depth.
2. The influencing factors of public service facility configurations are key forces that govern their construction [49]. Most existing studies showed that Christianity tends to consider population density and accessibility in the selection of missionary places [50]. However, the results of this study show that although they both exert some influence, it is weaker than that of population mobility, age structure, facility support, and the distribution of jobs-housing space. There is a clear circled spatial differentiation of these factors. Further, it shows that the socio-spatial relationship of the population and the provision of public goods have gradually replaced population density and transportation as the main drivers of location choice. There are three main reasons for this result. First, according to the large population migration between urban and rural areas in the early period, religion would spread along the population migration path based on the need for missionary work, so the layout of religious places was consistent with construction during that period. However, when urbanization entered the middle or later stages, the incremental development of cities slowly began to stagnate. On the one hand, there is significant social spatial differentiation within cities, and on the other hand, rural areas are slowly shrinking, which is the reason why the influencing factors of the space of religious places have changed. Secondly, in related research fields, the previous research methods lack quantitative models and often focus solely on field investigation. Different research methods are based on different data, so the difference of research technology is also one of the reasons for this phenomenon. Finally, different countries and cities often have divergent religious belief policies, religious categories, and worship patterns among other things. This study is based on typical cases in China, and the research results can be generalized to other Chinese regions to some extent. For wider applicability, a large number of case studies are needed in future.

### 5.2. Suggestions

In order to meet the needs of different religious people, the real value of religious places ought to take into account the practical needs of the religious bodies. However, due to the special nature of facilities and the mobility of the population, the traditional way of graded support and index setting is no longer applicable. It is now necessary to avoid structural problems such as an “imbalance” or “mismatch” between the supply and demand of Christian activity places and the believing community.

The spatial representation of religious places not only portrays the boundaries between differentiated groups, it also tests the government’s response to urban cultural and religious governance and related spatial development. Urban planning, as a value guide and macro-regulation of public policy, needs to be aware of the spatial polarization and residential segregation of religious groups that are triggered by the spatial differentiation of urban society, the relationship between equity and efficiency, and social heterogeneity and spatial homogeneity. Furthermore, socio-cultural elements such as religion should be incorporated into the modern urban governance system based on a full understanding of urban development and cultural governance in China. Religion, as well as social classes, are indispensable to urban multicultural spaces and places. Therefore, to realize the communities among different socio-cultural groups, this study examined the spatial differentiation of religious places from the perspective of the socio-spatial dialectic relationship, which is a refined theoretical guideline for religious governance to move towards place-specific and categorical approaches.

### 5.3. Outlook

The results of the GWR model in this study better explain the inner mechanism of spatial differentiation in the case of Christian activity places, but there are many influencing factors that cannot be considered in an integrated manner due to the difficulty of data acquisition. Moreover, the microscopic between religious people and places has been inadequately explored in terms of behavioral patterns and subjective awareness. This will be a key direction for future research.
